# Energy Restriction and Colorectal Cancer: A Call for Additional Research

**DOI:** 10.3390/nu12010114

**Published:** 2020-01-01

**Authors:** Maria Castejón, Adrian Plaza, Jorge Martinez-Romero, Pablo Jose Fernandez-Marcos, Rafael de Cabo, Alberto Diaz-Ruiz

**Affiliations:** 1 Nutritional Interventions Group, Precision Nutrition and Aging Program, Institute IMDEA Food (CEI UAM+CSIC), Crta. de Canto Blanco nº 8, E-28049 Madrid, Spain; mariacastejon1991@gmail.com (M.C.); decabora@grc.nia.nih.gov (R.d.C.); 2 Bioactive Products and Metabolic Syndrome Group-BIOPROMET, Precision Nutrition and Aging Program, Institute IMDEA Food (CEI UAM+CSIC), Crta. de Canto Blanco nº 8, E-28049 Madrid, Spain; adrian.plaza@imdea.org (A.P.); pablojose.fernandez@imdea.org (P.J.F.-M.); 3 Molecular Oncology and Nutritional Genomics of Cancer Group, Precision Nutrition and Cancer Program, Institute IMDEA Food (CEI, UAM/CSIC), Crta. de Canto Blanco nº 8, E-28049 Madrid, Spain; jorge.martinez@imdea.org; 4 Translational Gerontology Branch, National Institute on Aging, National Institutes of Health, 251 Bayview Boulevard, Baltimore, MD 21224, USA

**Keywords:** energy restriction, colorectal cancer models, metabolism

## Abstract

Colorectal cancer has the second highest cancer-related mortality rate, with an estimated 881,000 deaths worldwide in 2018. The urgent need to reduce the incidence and mortality rate requires innovative strategies to improve prevention, early diagnosis, prognostic biomarkers, and treatment effectiveness. Caloric restriction (CR) is known as the most robust nutritional intervention that extends lifespan and delays the progression of age-related diseases, with remarkable results for cancer protection. Other forms of energy restriction, such as periodic fasting, intermittent fasting, or fasting-mimicking diets, with or without reduction of total calorie intake, recapitulate the effects of chronic CR and confer a wide range of beneficial effects towards health and survival, including anti-cancer properties. In this review, the known molecular, cellular, and organismal effects of energy restriction in oncology will be discussed. Energy-restriction-based strategies implemented in colorectal models and clinical trials will be also revised. While energy restriction constitutes a promising intervention for the prevention and treatment of several malignant neoplasms, further investigations are essential to dissect the interplay between fundamental aspects of energy intake, such as feeding patterns, fasting length, or diet composition, with all of them influencing health and disease or cancer effects. Currently, effectiveness, safety, and practicability of different forms of fasting to fight cancer, particularly colorectal cancer, should still be contemplated with caution.

## 1. Colorectal Cancer Overview

An estimated 18.1 million new cancer cases and 9.6 million cancer deaths occurred worldwide in 2018. Among them, colorectal cancer (CRC) ranked third for incidence (10.2%, with 1.8 million new cases) and second for mortality (9.2%, with 881,000 deaths) [1,2]. Since 2000, a decline of the incidence and mortality rate of CRC has been observed, and is concomitant with a 5-year survival rate of 64.4% based on registries from Surveillance, Epidemiology, and End Results Program [SEER, 2009–2015] [3]. Progression of CRC is influenced by geography, human development index, age, genetic, environmental, and lifestyle factors [4]. Since aging is the major risk factor for all chronic diseases, including cancer, the population most frequently diagnosed with CRC is between 65–74 years old (SEER, 2012-2016) [5]. Importantly, an alarming increase of CRC in the population under the age of 55 has also recently been detected [4]. Besides age, inherited genetic syndromes, such as Lynch syndrome (hereditary non-polyposis colorectal cancer), familial adenomatous polyposis, and MutY DNA Glycosylase (MUTYH)-associated polyposis, are considered non-modifiable risk factors for CRC [6]. The prevalence of obesity, metabolic syndrome, non-alcoholic fatty liver disease (NAFLD), and other risk factors, such as alcohol consumption, smoking, physical inactivity, or diet rich in red and processed meat, also play a role in the pathogenesis of CRC [1,6,7]. On the other hand, evidence from epidemiological studies reveal that protective nutrition may reduce CRC incidence (reviewed in [8]). These nutritional practices include diets rich in fruits and vegetables, fiber, folate, calcium, garlic, dairy products, vitamin D and B6, magnesium, and fish [8].

Clinical manifestations of CRC are categorized in five stages (O, I, II, III, and IV). These stages determine treatment and prognosis, and are based on histopathological features, the degree of bowel wall invasion, lymph node spreading, and the appearance of distant metastases [9]. Early stages are often asymptomatic or concomitant with non-specific symptoms (i.e., loss of appetite or weight loss, anemia, abdominal pain, or changes in bowel habits) [8]. Later stages are concomitant with dissemination of cancer cells to the lymph system or other organs in the body. In this scenario, screening colonoscopies aimed at early diagnosis are recommended to start at the age of 45–50 years, a strategy that has contributed to the overall reduction of CRC incidence and mortality. Comprehensively, colorectal cancer diagnosed in adults aged 85 and older is often associated with a more advanced stage, with 10% less likelihood to be diagnosed at a local stage when compared with patients diagnosed at the age of 65 to 84 [10]. The most relevant mechanisms of CRC carcinogenesis identified to date include genetic chromosomal instability, microsatellite instability, serrated neoplasia, specific gene signatures, and specific gene mutations, such as APC (Adenomatous Polyposis Coli), SMAD4 (SMAD Family Member 4), BRAF (v-raf murine sarcoma viral oncogene homolog B), or KRAS (Kirsten rat sarcoma viral oncogene homolog). These mechanisms have been extensively described elsewhere [11,12]. Recent advances in technology for the analysis of body fluids (i.e., cell-free DNA and circulating tumor cells), epigenetic signatures (i.e., microRNAs, 5′-Cytosine-phosphate-Guanine-3′ (CPG) island methylator phenotypes, etc.), and microbial and immune elements are also uncovering distinctive prognostic biomarkers of CRC (reviewed in [2,11]). Further research aimed at the identification of unique CRC markers and their correlation with the behavioral and progression of CRC will be essential to personalize treatment and further reduce the rate of incidence and mortality of CRC.

## 2. Energy Restriction Overview

Energy restriction (ER) refers to dietary strategies in which energy intake is manipulated by inserting periods of time when calorie intake is reduced. Multiple aspects of dietary eating patterns, such as the timing or distribution of daily energy intake, meal frequency, or fasting length between meals, also influence energy and macronutrient intake, playing a central role in health [13,14]. The most popular forms of ER comprise continuous energy restriction (CER, also known as caloric or calorie restriction (CR)) and intermittent energy restriction (IER), which includes several dietary interventions such as intermittent fasting, periodic fasting, alternate day fasting, fasting-mimicking diet (FMD), or time restricted feeding [14]. CER involves a daily reduction of 20–40% of the total calorie intake, whereas IER requires the alternance of periods of severe or complete fasting with periods of greater energy consumption (refeeding), with or without reduction in the total amount of calories. Both nutritional strategies have shown physiological benefits, such as reduced body weight and inflammation, improved circadian rhythmicity and insulin sensitivity, autophagy, stress resistance, and modulation of the gut microbiota [13,14]. Despite these benefits, few comparative studies between CER and IER have been performed to date. In obese or overweight humans, these studies evidence similar effectiveness for body weight loss [15,16,17,18,19,20,21,22,23,24,25], with slightly better outcomes for IER regimens with regards to fat-free mass retention [16], fat mass loss, insulin sensitivity [24,25], postprandial lipemia [15], adherence, blood glucose, and anthropometric and lipid parameters [23]. It should be noted that these studies were performed with relatively small groups and for short periods of time, mostly ≤26 weeks. Therefore, larger and longer studies are pending to confirm these results. In any case, there is a general consensus in the field that both types of ER are similarly effective for weight loss and improvement of insulin sensitivity.

In many animal models ranging from yeasts to mice, ER has consistently been shown to extend lifespan [26]. In rhesus monkeys, CER delayed the appearance of age-related diseases and had a partial beneficial effect on total lifespan, indicating that CER also delays aging in non-human primates [27]. In humans, the longest nutritional trial conducted to date, the CALERIE (Comprehensive Assessment of Long Term Effects of Reducing Intake of Energy) study, subjected young non-obese human volunteers for 2 years to 25% CER in their phase 2 study group. In the absence of unsafe or detrimental effects, long-term CER reduced basal metabolic rate and metabolic syndrome score, and improved multiple systemic markers for cardiometabolic health, including reduced LDL-cholesterol, total cholesterol to HDL-cholesterol ratio, systolic and diastolic blood pressure, C-reactive protein, leptin, fasting insulin, insulin sensitivity index, thyroid hormones T3 and T4, nighttime core body temperature, and markers of oxidative damage (urinary F2-isoprostane); and increased adiponectin levels [28,29]. These results, although not conclusive, indicate that ER in humans can have an anti-aging effect, similar to that observed in all other animal models tested. Safe IER alternatives (i.e., alternate day fasting, time restricted feeding, or FMD) to CER have also shown to improve molecular markers of aging in healthy individuals [30,31,32]

## 3. Energy Restriction in Oncology

The fact that ER can delay cancer in animal models was first described in the 1980s [33]. Subsequent studies in mice or rats [34] have shown that CER inhibited spontaneous neoplasias in p53-deficient mice [35]; chemically-induced mammary [36], liver [37], or bladder [38] tumors; or radiation-induced tumors [39]. IER has been shown to prevent tumor formation in several mouse and rat models [40], including MMTV (mouse mammary tumor virus)-induced mammary tumors [41,42,43,44,45]; p53-deficient mice [46]; xenografted lung, ovarian, and liver tumor cell lines in nude mice [47]; and prostate tumor models [48,49]. Some reports, however, did not detect any protection in mice by IER from spontaneous mammary [50,51,52] or prostate [53,54] tumors, and others even showed increased tumor incidence with IER in chemical models of colon [55] or liver [56] cancers. For a more dedicated revision of these interventions in animal models, refer to [57]. Remarkably, in these last cases, IER began several days after the chemical insult was induced, suggesting that the precise timing of IER can be of great importance. Importantly, CER reduced the spontaneous appearance of cancer in rhesus monkeys [27].

In addition to a cancer-preventive effect of ER, more recent reports have consistently shown that ER can enhance the anti-tumor effect of chemotherapy, the standard therapy for most tumors [58]. In particular, fasting in mice with xenografted tumors of different origins enhanced the anti-tumor effects of several tyrosine kinase inhibitors [59] or other drugs [60], of temozolomide or radiation in glioblastoma xenografts [61], and of gemcitabine in pancreas xenografts [62]. Of note, 50% calorie restriction in glioblastoma-xenografted mice did not reproduce the cisplatin-sensitizing effects of fasting [63]. Apart from ER, fasting mimetic compounds have also been described to enhance chemotherapy effectiveness, as happened with the autophagy inducer hydroxycitate [64] or with the so-called fasting-mimicking diets [65]. In these last two reports, ER or ER mimetics enhanced chemotherapy efficacy by reducing the recruitment of immunosuppressing regulatory T cells and promoting the recruitment cytotoxic CD8+ cells to the tumor, suggesting for the first time an immunological component in the ER-mediated chemotherapy enhancement.

**Figure 1:**
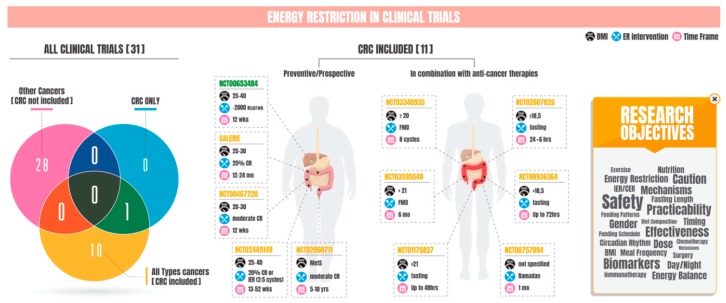
Energy restriction as a potential therapy for colorectal cancer. A Venn diagram (left side) is employed to categorize clinical trials on energy restriction (ER) and cancer by tumor type. Detailed features of the clinical trials on colorectal cancer are amplified in the central panel of the figure. Importantly, implementation of energy restriction for cancer prevention purposes is mostly carried out in overweight and obese populations. The word cloud (right side) shows essential variables or factors that impact the response to energy restriction, as well as necessary precautions that need to be contemplated for the use of energy restriction in oncology. Note: CR = caloric restriction; CRC = colorectal cancer; IER = intermittent energy restriction; FMD = fasting-mimicking diet; ER = energy restriction; BMI = body mass index; MetS = Metabolic Syndrome; CALERIE ((Comprehensive Assessment of Long term Effects of Reducing Intake of Energy) is composed of three different clinical trials (NCT00099151, with BMI 25–30; NCT00427193, with BMI ≥ 22 and <28; and NCT00099099, with BMI 25–30).

Anther very relevant beneficial effect of fasting during chemotherapy administration is the reduction in toxicity, which was first described in several mouse models in what was termed “differential stress resistance (DSR)” [66,67]. ER downregulates intracellular myogenic signaling, slows metabolism, increases mitochondrial efficiency, and reduces oxidative stress, leading to cell cycle arrest and increased resistance to stress [28]. In cancer cells, uncontrolled activation of growth signals and loss of antiproliferative signals by mutations in tumor suppressor genes impairs ER-induced stress protection [68]. Therefore, fasting protects from chemotherapy, radiotherapy, or tyrosine kinase inhibitor (TKI) toxicity in healthy cells but not tumor cells, leading to increased efficiency of these agents, known as “differential stress sensitization” (DSS) [59,60,61,66,69,70,71,72]. Most importantly, this protective effect of fasting was reproduced in two randomized clinical trials with human patients with breast [73] or breast and ovary [74] tumors. Two other reports mixing different tumor types reported that fasting for up to 72 h was safe and feasible in combination with chemotherapy [75,76]. Finally, feasibility and adherence for a completed clinical trial testing reduced calorie intake prior to surgical prostatectomy in prostate cancer patients was published, although no data on tumor markers is available yet [77]. These promising findings paved the way for several new ongoing clinical trials aimed at applying ER-based strategies for the prevention of cancer development, improvement of cancer chemotherapy effects, and reduction of chemotherapy-associated toxicity (see Table 1 and Figure 1, left side), although no new publication is available yet.

## 4. Fundamental Metabolic and Systemic Adaptations Induced by Energy Restriction in Oncology

**Figure 2:**
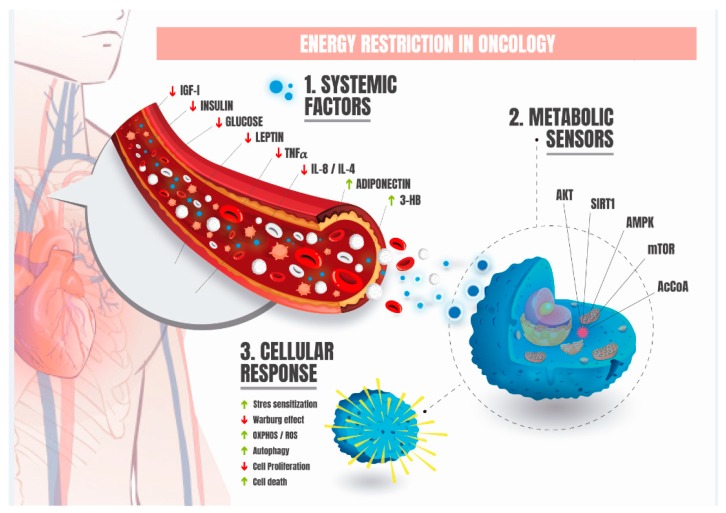
Overview of the fundamental characteristics of energy restriction in oncology. (1) Systemic factors: health-associated circulating factors modulated by energy restriction with potential implications for cancer prevention or treatment are depicted. (2) Metabolic sensors: intracellular nutritional sensors regulated by ER with the capacity to synchronize the nutritional status and levels of systemic factors with the cellular response. (3) Cellular response: activation of metabolic sensors triggers transcriptional programs and cellular physiological responses that play major roles in the context of carcinogenesis. Note: IGF-1 = insulin-like growth factor 1; TNFα = Tumor Necrosis Factor alpha; IL = Interleukin; 3-HB = 3-Hydroxybutyrate; AKT = Protein Kinase B; SIRT1 = Sirtuin 1; AMPK = AMP-activated protein kinase, mTOR = mammalian target of rapamycin; AcCoa = Acetyl coenzyme A.

ER induces a plethora of physiological and biochemical changes. At the organismal level, ER is associated with reduction of body weight, central adiposity, chronic inflammation, enhancement of insulin sensitivity, and improvement of metabolic flexibility. Metabolic flexibility is defined as the organismal capacity to respond and adapt fuel selection between glucose, fatty acids, and proteins to the physiological state, metabolic demand, or energy or nutrient availability. Comprehensively, metabolic inflexibility plays a major role in the transition between health and disease and is correlated with an increased risk of certain types of cancers [78,79]. Systemically, ER also modulates numerous circulating factors (Figure 2), including growth factors and energy-balance-related hormones, many of which have previously been associated with cancer development and can help explain the anti-cancer effects of ER.

Insulin, insulin-like growth factors 1 and 2: Calorie restriction and intermittent fasting decrease the levels of fasting blood insulin [80,81,82]. As for the related hormones insulin-like growth factors 1 and 2 (IGF-I and IGF-II), intermittent fasting and protein-restriction, but not calorie restriction, were shown to reduce IGF-I levels [83]. Importantly, high levels of fasting blood insulin have been associated with increased risk of cancer in general [84], and more precisely, of breast [85], pancreas [86], and prostate [87,88] cancers. For colorectal carcinoma, there is conflicting evidence; while some studies detected increased risk of CRC in patients with high fasting blood insulin [89], other studies failed to detect this association [90,91,92]. Likewise, whereas some studies associate IGF-I or IGF-II with increased risk of colorectal carcinoma [93,94] and several other types of tumors [95,96,97], other reports failed to detect this association [98,99,100,101,102,103,104]. 

Blood Glucose: Fasting blood glucose is decreased by both calorie restriction [80] and by periodic fasting [105]. In turn, high fasting blood glucose levels are associated with increased risk of prostate [106], pancreas [107], lung [108], or stomach [109] cancers. More relevant for this revision, increased blood glucose levels are significantly associated with increased CRC risk in several studies [110,111,112].

Leptin: Leptin is a cytokine secreted by the adipose tissue (also called adipokines). In particular, leptin is released shortly after feeding and regulates satiety signals in the central nervous system. Leptin is decreased with calorie restriction [113] and intermittent fasting [114,115], and is increased with obesity due to the development of leptin resistance [116]. Increased blood leptin is associated with increased risk of developing breast [117] or thyroid [118] cancer. Interestingly, certain polymorphisms in the leptin gene have been associated with increased CRC in women [119]. However, no changes in serum leptin were found in CRC patients [120], and in fact, increased expression of leptin and leptin receptor in CRC samples was associated with better prognosis [121]. Therefore, the role of leptin in CRC is still unclear.

Adiponectin: Adiponectin is another adipokine that is decreased in obese individuals and increase with weight loss [122]. Decreased serum adiponectin has been associated with higher risk of several types of cancer [123], including breast cancer, especially in postmenopausal women [124,125]; endometrial cancer, especially in younger women [126] with reported but controversial anti-cancer effects; gastric cancer [127]; prostate cancer [128]; and some types of myeloblastic leukemias [129]. As for CRC, several reports have shown an inverse association between serum adiponectin and CRC risk [130,131], although other reports failed to detect this association [132].

Ketone bodies: Ketone bodies are generated in the liver from fatty acids when fasting, constituting the main energy source for many organs [133]. Many preclinical models have shown anti-tumoral effects of ketogenic diets [134]. In humans, only case reports or pilot studies have been performed to date, but many of them showed very positive effects of this type of diet, supporting further research in this direction [134].

Systemic inflammatory cytokines: Serum levels of several cytokines, including TNFα, IL-8, and IL-4, among others, are elevated in patients with CRC, and some studies have suggested that increased plasma levels of these cytokines may have a prognostic value [135]. Nutritional ER interventions are shown to lower the levels of circulating proinflammatory cytokines in humans and preclinical models [136,137,138], representing one of the ER-associated mechanisms against tumor development and growth.

## 5. Fundamental Cellular and Molecular Adaptations Induced by Energy Restriction in Oncology

Central mechanisms of ER have been investigated and reviewed in recent decades, many of them with implications for fundamental aspects of the well-defined hallmarks of cancer [139,140]. Herein, we focus on reviewing the main signaling pathways involving intracellular energetic sensors and cellular metabolic responses that influence growth and survival in cancer cells, and how these processes can be modulated by ER (Figure 2).

Warburg effect and reactive oxidative species (ROS): uncontrolled growth and proliferation impose a high energetic demand that needs to be supported by a metabolic shift in cancer cells. Glucose and glucogenic amino acids are used to fuel aerobic glycolysis and its end product lactate, at the expense of mitochondrial oxidative phosphorylation, a process known as the Warburg effect [68]. These unique metabolic features are also recognized as promoters of enhanced resistance to chemotherapeutic agents in these cells [141,142]. 

Cancer cells also exhibit deregulation of reactive oxygen species (ROS) homeostasis. Imbalance between ROS production and detoxification, likely by impaired antioxidant capacity, amplifies oxidative protein, lipid and DNA damage, genomic instability, and DNA mutations, thus contributing to cancer development and progression. In this line, genetic ablation of the master regulator of anti-oxidative defense, NRF2, or its downstream intermediaries (i.e., NQO1), favors tumor growth in chemical-induced models of tumorigenesis [143,144]. Redox signaling and endoplasmic reticulum stress (ERS) constitute other pro-tumorigenic mechanisms driven by ROS. It has been reported that ROS-mediated inhibition of the tumor suppressor PTEN hyperactivates AKT and mTORC1, therefore contributing to deregulated control of energy metabolism and neoplastic proliferation (reviewed in [145]). Likewise, chronic activation of the ERS sensors (IRE1α, ATF6α, and PERK) promotes unfolded protein response (UPR), favoring cancer cell autonomous and non-autonomous processes that lead to an immunosuppressive and pro-tumorigenic microenvironment [146,147]. Importantly, excessive ROS surpassing the antioxidant capacity threshold may result in severe ERS, unsustainable macromolecular damage, and cellular death [146,148]. In fact, increased susceptibility to ROS- and ERS-inducing cell death is documented for cancer cells, representing a window for multiple ROS- and ERS-driven anti-cancer therapies oriented to either increase the rate of OXPHOS or ROS production [149], ERS induction [150,151], or to inhibit ROS scavenging [149]. In the context of CRC, Rubio-Patiño and colleagues showed in different mouse cancers models of CRC that mice fed a low-protein diet prompted the induction of IRE1α and RIG1 signaling downstream of ERS, resulting in increased levels of cytokines, effective assembly of the immune response, and reduced colorectal tumor growth [146]. In another study, stimulation of the transcription factors ATF6 and XBP1 downstream of PERK signaling reduced proliferation of colorectal cancer cells [152]. Likewise, ROS-based therapies have been extensively investigated in CRC [148]. 

In this scenario, protective benefits of ER against cancer may rely on several mechanisms. On one hand, implementation of ER strategies may induce tumor-cell-specific ERS and cell death, as reported for a low-diet protein. On the other hand, ER is shown to consistently induce mitochondrial biogenesis and to stimulate OXPHOS [153]. Restriction in glycolysis and glutaminolysis concomitant with increased fatty acid lipolysis and ketogenesis also represents an ER-dependent metabolic mechanism with anti-cancer properties [140]. Bianchi and colleagues reported that complete fasting (48 h) reverses the Warburg effect, increases OXPHOS, and promotes ROS generation and apoptosis [69]. This metabolic reprogramming is partly mediated by the activation of the transcription factor PPARα [140]. Indeed, exogenous administration of PPARα agonists has been shown to inhibit cell growth in CRC cell lines [154].

AKT/mTOR pathway: Binding of extracellular growth factors such as IGF-1 or insulin to tyrosine kinase (TK) receptors triggers the activation of mitogen signaling pathways through multiple downstream intermediaries, including PI3K, AKT, and the molecular machinery responsible for ribosome biogenesis and protein translation (mTORC1, 4EBP1, S6K1, RPS6). These pathways are well-recognized as hallmarks of cancer and regulate glycolysis, cell growth, and proliferation, as well as resistance to apoptosis, angiogenesis, and metastasis [68]. In this scenario, targeting PI3K/AKT/mTOR has become of high interest in the field of cancer and CRC, with a number of anti IGF-1 antibodies and tyrosine kinases or AKT/mTOR inhibitors being tested in numerous clinical trials [155,156,157]. Likewise, the PI3K/AKT/mTOR pathway is central in the development and progression of CRC [157,158]. Additionally, mTOR is found upstream of many genetic alterations responsible for CRC carcinogenesis [159], and multiple CRC cells lines contain mutations of the PI3K/PTEN/AKT pathway [157]. As described above, reduction of circulating proliferative signals (i.e., insulin, glucose, IGF-1, and other GFs) is strongly achieved by ER, and a consequential downregulation of intracellular mitogenic signaling in tumors has also been observed [160]. Combined effects of systemic and intracellular processes on proliferative pathways constitute an essential advantage prompted by ER against cancer.

AMPK, autophagy, and SIRT1: The sensing capacity of growth signals and nutrient availability is essential to trigger adaptive metabolic responses favoring tumor growing. AMPK and SIRT1 are two crucial nutrient sensors modulated by ER that influence cell metabolism and energetic homeostasis, and therefore are deeply implicated in fundamental cell processes, such as proliferation, autophagy, or apoptosis [140]. AMPK senses the intracellular levels of ATP/AMP to adjust catabolic or anabolic pathways in response to oxidative stress and energy demand. AMPK activation inhibits the mTOR pathway, promotes fatty acid oxidation [161], disables the Warburg effect by restricting anaerobic glycolysis and promoting mitochondrial respiration [162], and plays an essential role in maintaining redox homeostasis by balancing NAD(P)H/NAD(P)^+^ ratio levels [163,164]. Since cancer is concomitant with a metabolic status of a high energetic demand, AMPK activators are suitable targets for cancer therapeutics [165,166]. ER-based strategies decrease ratios of ATP/AMP, which translates into activation of AMPK and phosphorylation-mediated activation of mTORC1 inhibitors, contributing to reduced cell growth and proliferation [167]. It should be taken into consideration that although AMPK is traditionally considered as a tumor suppressor gene, higher levels of AMPK have been associated with chemoresistance in many cancer types [168], and elevated expression of AMPK correlates with some clinicopathological factors of poor prognosis for breast cancer [169]. Jeon and colleagues revealed that AMPK activation is surprisingly connected to NADPH homeostasis and enhanced stress resistance to promoting cancer cell viability under certain conditions, such as hypoxia and metastasis [170]. Thus, increasing evidence suggests that AMPK may have cancer-promoting roles. This discrepancy has been also documented for CRC cancer. Simultaneous activation of AMPK and MAPK signaling was associated with better prognosis in a study with 718 CRC cancer samples [171]. On the other hand, Wang et al. demonstrated that high levels of AMPKα1 correlates with poor prognosis in CRC patients [172]. Since AMPKα1 confers survival advantages, inhibition of AMPKα1 promotes CRC cell death, likely by attenuating glutathione metabolism [172]. Moreover, AMPK has been recently reported to be directly implicated in CRC relapse and metastasis, since it promotes the survival of cancer stem cells by reprogramming their metabolism [173]. In this scenario, understanding the double-edged sword of AMPK in the context of cancer, and particularly CRC, remains a challenge. 

The activation of AMPK by ER induces cancer cell autophagy, a fundamental process in recycling unnecessary or dysfunctional cellular components to sustain mitochondrial metabolic function and energetic homeostasis [140,174]. Under nutrient scarcity conditions, activation of AMPK also induces SIRT1 activity [175]. Likewise, autophagy is also stimulated by SIRT1. The family of SIRTs proteins sense NADH/NAD^+^ ratios and exert NAD^+^-dependent deacylase or mono-ADP-ribosyltransferase activities, playing central roles in metabolism, transcription, apoptosis, inflammation, and stress resistance in the context of aging and cancer [139].

ER is shown to increase NAD^+^ levels, stimulate SIRT1, and promote autophagy [176,177]. Therefore, preventing toxic accumulation of protein or organelles and oncogenic signaling through autophagy represents another defensive influence mediated by ER-based strategies against cancer and CRC [178], although different sensitivities for autophagy inhibition in CRC have been described [179]. Importantly, contradictory implications for autophagy and SIRT1 in carcinogenesis and CRC are also found, with oncogenic or tumor suppressive functions depending on the cancer type and the particular background or tumor microenvironment [174,178,180,181]. A systematic meta-analysis carried out by Zu et al. also revealed that SIRT1 expression correlates with depth of invasion and predicts a poor overall survival [182]. Therefore, understanding the roles of autophagy and SIRT1 in specific contexts, tumor microenvironments, and different stages is essential to apply autophagy- and SIRT1-driven strategies for the prevention of CRC development and reduction of CRC malignancy and progression.

## 6. Chemical-Induced Models of CRC: Impact of Energy Restriction

**Figure 3:**
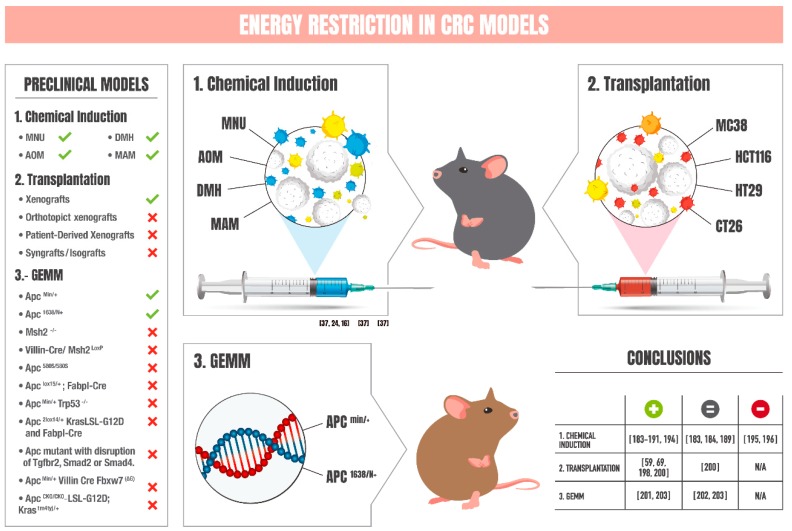
Energy restriction strategies implemented in preclinical models of colorectal cancer. The main preclinical models used for the study of colorectal cancer are shown in the left column and described elsewhere [189]. Within this column, green ticks indicate those models in which interventions based on energy restriction have been applied. These models are illustrated in the central panel of the figure, with the main conclusions (positive, negative, or unchanged) of the studies summarized in the adjacent table (bottom right). Note that for several studies, the positive or unchanged response against carcinogenesis depends on multiple factors that influence energy restriction, such as diet, dose of restriction, or onset of the intervention. GEMM = Genetically Engineered Mouse Models; MNU = N-methylnitrosurea; MAM = methylazoxymethanol acetate; AOM = azoxymethane; DMH = dimethylhydrazine; APC = Adenomatous Polyposis Coli.

A common method to induce intestinal tumorigenesis in animal models consists of the inoculation of certain types of drugs, such as methylazoxymethanol acetate (MAM), N-methylnitrosurea (MNU), azoxymethane (AOM), or 1,2-dimethylhydrazine (1,2-DMH). The vast majority of studies reveal protective effects of ER strategies for tumor development and tumor growth when using these methods (Figure 3). In male Sprague–Dawley rats, sustained 25% dietary restriction (DR) intervention conferred advantageous effects after a single inoculation with MAM [183,184]. Continuous reduction (30%) of energy intake significantly lessened tumor number and tumor size in male F344 rats, assessed at 32 weeks after a second subcutaneous injection of AOM [185]. In this case, rats were challenged with a high-fat diet (HFD) to enhance tumor progression. In another study in rats, 5 intraperitoneal injections (once a week for 5 weeks) of AOM beginning at the age of 10 weeks were followed by 20 weeks of CR regimen set at 30% reduction of daily calories. This strategy was sufficient to diminish the number of colon tumors and was concomitant with reduction of inflammatory cytokines, IGF-1, and cell proliferation, the latter assessed via immunohistochemistry for proliferating cell nuclear antigen (PCNA) [186]. In this study, CR also increased levels of IGFBP-3 and induced cellular apoptosis. Importantly, several miRNAs (mir-425, mir-196, mir-155, mir-150, mir-351, mir-16, let-7, mir-34, and mir-138) were found differential to be expressed between interventions, providing insights into the mechanistic effect of the protection against carcinogenesis induced by CR in this model [186]. Tumor incidence and tumor number were also significantly reduced in the outbred ICR (Institute of Cancer Research) mice strain injected with AOM and fed over 13 weeks to 20% CR when compared with ad libitum (AL) controls. Colon tumor protection was concomitant with reduction and augmentation of mRNA levels of the anti-apoptotic Bcl-2 and proapoptotic Bax, respectively [187]. In another study, 40% of CR was sufficient to diminish the number of rat preneoplasic colonic aberrant crypt foci (ACF) only 5 weeks after DMH-treatment. When compared to AL regimen, CR was associated with reduction of colonic mucosa proliferation, reduction of cell cycle-progression genes (surviving and cyclin D1), and augmentation of all SIRT1-7 mRNAS [188].

The interplay between dietary composition and dosage of ER has also been investigated in chemically induced models of CRC. Male F344 rats were given 2-AOM injections once a week at the age of 7 weeks. Thereafter, rats were fed to 20% CR on a low-fat diet (LFD), or fed to 10%, 20%, or 30% CR regimen on a HFD for the next 32 weeks. Unexpectedly, 20% CR on LFD did not significantly reduce tumor incidence (i.e., % of animals with tumors) or tumor multiplicity (i.e., number of tumors per animal) when compared to LFD controls fed ad libitum (AL) [190]. In the context of an obesogenic diet, a significant dose response was observed, with chronic reduction of 20% and 30%, but not 10% HFD, exhibiting a strong cancer protection for the above-mentioned parameters. Tumor progression in these groups diminished to levels closely related to LFD-AL controls [190]. In another study, F344 rats were injected with AMO, fed AL for 11 weeks, and then separated into LFD and HFD in the presence or absence of 20% CR for another 12 weeks. At the end of the intervention, CR significantly diminished rat preneoplasic colonic aberrant crypt foci (ACF) in a diet-independent manner, with a positive correlation observed between daily energy intake and the number of ACF/colon [191]. A follow-up study from the same authors subjected F344 rats to AL regimen for 16 weeks after the injection with AOM. Thereafter, rats were fed for another 12 weeks with LF or HF diets in the presence or absence of CR. Although levels of ACF were reduced independently of fat content after 6 weeks of CR, the appearance of advanced ACF (higher crypt multiplicity) was also observed in the context of LFD but not HFD [192]. These studies clearly indicate that calories and dietary fat are both cancer-promoting factors [193,194], in agreement with epidemiological data supporting the association between energy balance and the prevalence of metabolic diseases (i.e., obesity, metabolic syndrome, or NAFLD) with colon cancer. Furthermore, they reinforce the notion that CR effectiveness may be influenced by dietary fat content and dose of energy reduction. In another study, the incidence of colonic carcinogenesis after administration of 1,2-DMH was significantly reduced in rats fed for 6 months to 40% less calories than rats given AL regimen. In this article, dietary composition varied between groups, with 4% and 13.1% of fat content of rats fed to AL or CR, respectively [195].

It should be noted that conflicting data reporting an enhanced tumorigenic effect mediated by CR implementation has also been published. In male Sprague–Dawley rats, unchanged tumorigenic response was reported between rats fed to AL or rats undergoing daily 25% dietary restriction after a single inoculation with MNU [184]. In a similar study, rats that were 25% continuously restricted from day 63 after MAM injection or rats undergoing 25% restriction every-other day from day 8 or day 31 after the injection showed no significant reductions of intestinal tumors when compared to AL controls [183]. Since in this study tumor protection was successfully achieved when 25% CER was implemented early on (from day 100), these data are in agreement with the current notion that beyond energy reduction, onset of treatment or feeding schedule represent important energy intake factors for tumor outcome [14]. In another study, rats exposed for 56 days to 40% of food deprivation after a single injection of DMH resulted in enhanced formation of early tumorigenic lesions, likely by suppression of serotonergic activity in colon tissue, which has been described to promote early steps of cancer development [196]. This study suggests that short periods of ER may not show the potential to protect against colon carcinogenesis. Fasting for 4 days was also shown to enhance growth of ACF in rat colon and rectum after exposure to AOM on the same day of refeeding. In this study, while fasting induced apoptosis and reduced cell division, refeeding resulted in higher percentage of S-phase cells, even after 2 days of AOM injection [197]. These studies, beyond implying cautiousness when applying ER strategies as anti-cancer therapies, highlight the need to acutely understand how tumor outcomes are influenced by multiple factors, such as method of induction, dose of CR, diet composition, feeding schedule, or the onset of ER intervention.

## 7. Transplantation Models of CRC: Influence of Energy Restriction

Transplantation models for the study of colon cancer have been extensively detailed by McIntyre et al. [189]. These models include: (1) xenografts, or subcutaneous injection of CRC cell lines (such as HCT116, HT29, CT26, or MC38) into immunosuppressed mice; (2) orthotopic xenografts, or injection of CRC cell lines into intestinal serosa in immunosuppressed mice; (3) patient-derived xenografts (PDXs), wherein tumor fragments are sutured from patients to immunodeficient mice; and (4) syngrafts or isografts, where tumor fragments are sutured from mice to genetically identical, inbred, immunocompetent mice. Among these alternatives, only a few studies using xenografts have examined the anti-cancer effects of ER (Figure 3). In 2013, Harvey et al. implemented 30% CR in female C57BL/6 mice for 21 weeks prior to injection of tumorigenic MC38 cells. At 24 days after injection, CR-fed mice exhibited lower tumor volume concomitant with reduced body weight, body fat, serum IGF-1, leptin, and insulin. Tumorigenic gene signature was characterized by reduced expression of inflammatory and cancer-related genes, such as IL6, IL-1B, TNFα, or cyclooxygenase 2 [198]. More recently, short-term starvation (STS, consisting of 48 h of complete food deprivation with free access to water) implemented at 1-week intervals was sufficient to reduce tumorigenesis in BALB/c mice after injection of CT26 cells [69]. Combination of STS with the chemotherapy agent oxaliplatin (OXP) potentiated the effects on the progression of colorectal tumors. Increased tumor chemosensitivity was associated with reduction of the “Warburg effect” in tumoral cells. In this regard, reduced glycolytic rate and increased oxygen consumption or oxidative phosphorylation was concomitant with failure to generate ATP, likely due to deficiency in glucose and amino acid availability, which resulted in oxidative damage and cellular apoptosis [69]. STS-mediated reduction of tumor volume has been also reported to be effective after injection of HTC116 cells in BALB/c mice [59]. Likewise, combination of STS with Regorafenib, a multi-target tyrosine kinase inhibitor (TKI), potentiated the anti-cancer effects, likely by improving the TKI-mediated inhibition of transcription driven by mitogen-activated protein kinase [59]. Alternate day fasting, consisting of alternate days of complete fasting and ad libitum diet on non-fasting days, has been also shown to be protective against cancer progression induced by CT26 cells [199]. In this article, suppression of M2 polarization of tumor-associated macrophages was reported to be a fundamental process induced by fasting to reduce colorectal tumor growth, likely mediated by a deficit of adenosine in the tumor microenvironment [199]. The effect of dietary composition against colorectal tumorigenesis has also been studied using MC38 cells. Obese mice (where obesity was induced by high-fat (HF) diet consumption) were kept for 15 weeks on a low-carbohydrate diet (LC), a high-carbohydrate diet (HC), or a HC diet in combination with 30% of CR (HC-CR). Compared to control HF group, LC and HC alone were not sufficient to reduce tumor growth in obese mice, despite the lower body weight and lower levels of IGF-1 and leptin in the HC group. Contrary, HC-CR group exhibited the longest time to palpable tumors, lower percentage of mice with tumors, and reduced tumor size [200]. Overall, the use of xenografts has demonstrated advantageous effects of ER in the protection against colon carcinogenesis. Nonetheless, these studies are carried out by subcutaneous implantation of CRC cells followed by the cellular and molecular analysis of the tumor at the primary site or location, which may not recapitulate fundamental processes of colorectal growth and progression, such as interaction between tumor cells, stroma, or the immune system. Since these models may not be fully representative of the pathophysiology of CRC, results must be interpreted with caution.

## 8. Genetically Engineered Mouse Models of CRC: Effects of Energy Restriction

Mouse models genetically engineered (GEMM) for the most frequent mutated genes in CRC are also available. A detailed description of the advantages and disadvantages for each model has been given elsewhere [189]. Examples of implementation of ER-based strategies have been published for three of these models (Figure 3). In 2003, Mai et al. found that implementation for 15 weeks at 40% CR to 9-week-old male Apc^Min^ mice resulted in 57% reduction of the frequency of intestinal polyps, concomitant with reduced levels of serum IGF-1 and leptin [201]. In another study, 15-week-old Apc^1638/N+^ mice underwent 40% of CR for 12 weeks. Importantly, formation of macroadenomas was significantly reduced in males fed to CR, but not in females. CR diet also failed to reduce body mass, adiposity, or serum levels of glucose, insulin, or leptin in females [202]. Despite no effect on these parameters, CR enhanced survival in females, a phenomenon that was not observed in males. In a third study, Apc^Min/+^ mice at the age of 5 or 10 weeks were fed a diet devoid of methyl donors (MDD) (i.e., folate, choline, methionine, and vitamin B12) [203]. Although no effect was observed in colon tumor formation or size in the colon, feeding with this MDD diet suppressed formation of small intestinal tumors when started at 5 but not 10 weeks of age. Since MDD group also reduced body weight, suggestive of reduced caloric intake, the authors assessed a second study including a pair-fed (PF) group with MDD. Of note, the PF group also showed lowered number of small intestinal tumors, although the anti-cancer effect was exacerbated in MDD group. Despite this few studies, the efficacy of ER-based interventions against colon carcinogenesis using GEMM remains to be explored. 

## 9. Energy Restriction and Clinical Trials in CRC

Only one clinical trial has been performed that specifically examines the effects of ER intervention for the prevention of CRC (NCT00653484; Table 1 and Figure 1). This study, with no available publications yet, aimed to identify beneficial effects of a 12-week dietary restriction intervention (−2000 kcal/week), with or without physical activity (+1000/−1000 kcal/week), in colorectal-carcinoma-predisposed healthy, overweight, or mildly obese individuals. Prevention of all-type cancers (CRC included) through energy restriction was assessed by the CALERIE trial (NCT00099151, NCT00427193, NCT00099099; Table 1 and Figure 1) and the Healthy Nutrition and Energy Restriction as Cancer Prevention Strategies (HELENA): a Randomized, Controlled Intervention trial (NCT02449148; Table 1 and Figure 1). The CALERIE study tested whether long-term CER in a population with body max index (BMI) between 22 and 30 improves risk factors of longevity in humans and prevents some age-related chronic diseases, including cancer. The HELENA trial implemented 50 weeks of continuous (20% of daily energy deficit) or intermittent caloric restriction, the latest in the form of “5:2 diet” (5 days/week at regular energy intake and 2 days/week at a ∼75% energy deficit) among 150 overweight or obese individuals. Based on the analysis of body composition, anthropometrics, adipose tissue gene expression, and metabolic biomarkers, the authors concluded that the effects of IER may be equivalent but not superior to CER for weight reduction and prevention of metabolic diseases [204]. Two other clinical trials (NCT02960711 and NCT00467220) aim to examine the effects of dietary restriction on the risk for cardiovascular disease and cancer; and to prevent age-related, chronic, non-communicable diseases, including cancer, respectively. In patients with any malignant neoplasm in which CRC could be comprised, six additional trials aim to determine the safety and feasibility of several fasting strategies, including the month of Ramadan (NCT00757094); short-term fasting for up to 72 h, mostly prior to chemotherapy (NCT01175837, NCT00936364, and NCT02607826); or 8 cycles of fasting-mimicking diet (FMD) (NCT03340935). The FMD is a patented plant-based, low-calorie, low-protein, and low-carbohydrate diet designed to mimic fasting while providing adequate micronutrients (vitamins, minerals, etc.) and minimize the burden of fasting [30]. Periodic cycles (5 days a month, 600 Kcal on day 1 followed by 300 KCal/day on days 2 to 5) of this diet followed by a normal feeding regimen promotes increased lifespan in humans [30]. A Phase II clinical study on FMD diet in patients undergoing breast and colorectal oncologic treatment is currently recruiting (NCT03595540; Table 1 and Figure 1). Except for the HELENA trial, publications from these trials are not available.

## 10. Future Directions

Convincing evidence demonstrates numerous physiological benefits associated with ER-based interventions. Enhancement of health and improvement of molecular markers of aging seem to translate well between model organisms and humans under different forms of ER. In the context of cancer, preclinical models have been useful tools to decipher organismal adaptations to fasting and the cellular and molecular responses that occur in cancer cells in response to nutrient scarcity. These findings have paved the way to conceive ER interventions as feasible strategies with the potential to prevent or delay the appearance of cancer and to reduce chemotherapy toxicity in the treatment of several types of cancer. In this revision, we have acknowledged 39 recent clinical trials using ER approaches for these purposes, with 13 of them still enrolling, recruiting, or active but not recruiting subjects (Table 1). Despite the fast growth of ER usage against cancer, clinical data from these studies is still not available, with only 4 research articles published to date (see Table 1); while promising, one should remain cautious about these interventions. Others have put forward concerns regarding weight-loss or reduction of protein intake reached with ER, which largely disagree with nutritional recommendations for cancer care and may aggravate age-associated risk of malnutrition or sarcopenia in cancer patients [205]. In this context, nutritional supplements or combination of ER with exercise may be reasonable approaches to minimize ER side-effects while maximizing ER advantages [206]. It must be stated that conflicting data reporting absence or even undesirable pro-carcinogenic effects in response to ER should not be underestimated in the race to translate these interventions to humans. For example, there was an increase in pulmonary colonization and metastasis in mice undergoing 30% CR, despite an evident reduction of growth of B16 melanomas at the primary site [207]. As suggested by García-Jiménez et al., starvation in the tumor micro-environment may promote a proliferative-to-invasive phenotypic transition in cancer cells, likely by phosphorylation of eIF2α, linking cell starvation and metastasis [208]. Compiled information from the Dutch population during World War II also insinuates that food restriction may even increase tumor risk in humans [209], even though this effect could be due to malnutrition in addition to the ER. Therefore, as of today, precaution should still be exercised in this field. In the context of CRC, it is our belief that a significant challenge lies ahead for the scientific community and should precede the conversion of ER interventions to the clinic. In preclinical models of CRC, we have accounted for 20 studies in which ER interventions are applied (12 for carcinogenic-induced models with chemicals, 5 for transplantation of CRC cell lines, and 3 using genetically modified mouse models of CRC; Figure 3). In our opinion, findings from this work are convincing. However, ER effects are not always universal [210,211]; the impact of uncharacterized modulators of the response to ER, such as sex, dose, meal frequency, timing, circadian rhythms, or length of fasting times in cancer, remains to be fully understood. Of note, only 1 clinical trial is exclusively targeting CRC cancer, with 10 other trials recruiting all-type cancers in which CRC is also comprised. Personalized nutritional practices in CRC patients may require special attention to avoid possible ER- or age-associated malnourishment or sarcopenia, since the mean age at diagnosis for this type of cancer is 67 years [3], and advances stages are usually concomitant with cachexia [212,213]. In closing, with this review we intend to stimulate further research to (1) address comparative ER interventions in CRC models, to (2) improve our understanding of the fundamental mechanisms underlying the favorable effects of ER in CRC, and to (3) use this new information to successfully develop personalized, effective, and safe ER interventions for treatment in CRC patients. 

## Figures and Tables

**Table 1:** Clinical trials on energy restriction (ER) and cancer. Clinical trials posted in the ClinicalTrials.gov public database including the keywords “diet”, “fasting”, “cancer”, “energy restriction”, “calorie restriction”, or “caloric restriction”. The list includes trials with a well-defined ER intervention, either as a preventive strategy or in combination with anti-cancer treatment (chemotherapy, radiotherapy, surgery, or a combination of these strategies). Note: QD = once daily; WCRF/AICR = World Cancer Research Fund/American Institute for Cancer Research; CLS = macrophage crown-like structures; FACT-G/O/P = Functional Assessment of Cancer Therapy-General/Ovarian/Prostate; HER2 = human epidermal growth factor receptor 2; ACSM = American College of Sports Medicine; NSCLC = non-small cell lung cancer; RECIST = Response Evaluation Criteria in Solid Tumors; DPP = Diabetes Prevention Program.

Clinical Trial Identifier	Clinical Trial Title	Study Objective	Tumor Type	Energy Restriction Type	Primary Outcome	Time Frame	Beginning	Associated Publication
NCT01535911	Pilot Study of a Metabolic Nutritional Therapy for the Management of Primary Brain Tumors	In combination with anti-cancer therapy	Glioblastoma	Energy-restricted ketogenic diet (ERKD) (metabolic nutritional therapy). Total calories consumed by each subject will be targeted to 20 to 25 kcal/kg/day. If the tumor has decreased in size or the size has remained the same then subjects will be continued on the ERKD for an additional 6 weeks and a repeat MRI will be obtained.	MRI imaging will be used to measure changes in brain tumor size. (Time Frame: 6 weeks after completion of radiation therapy). Results of the metabolic therapy will be assessed by comparing MRI images obtained at the beginning of the study with those after completion of radiation therapy and after an additional 6 weeks of metabolic therapy.	6 years	2012	doi:10.1186/s40170-015-0129-1
NCT01819233	A Feasibility Pilot Trial Evaluating Caloric Restriction for Oncology Research in Early-Stage Breast Cancer Patients	In combination with anti-cancer therapy	Stage 0–I breast cancer	Beginning 2–4 weeks after completion of lumpectomy, patients receive food diaries to complete for 7–10 days. Dietary counselors then give patients guidelines for dietary modifications to reduce caloric intake by 25% of their normal diet. Patients follow calorie-restricted diet for 10 weeks (2 weeks prior to radiation therapy, during 6 weeks of radiation therapy, and at least 2 weeks after radiation therapy). Patients undergo radiation therapy QD 5 days a week for 6 weeks.	Proportion of patients who are adherent to the diet restriction. (Time frame: up to week 12).	4 years	2013	
NCT03625635	Effect of a Clinical Nutrition Intervention Program on Body Composition, Metabolism, and Antioxidant Activity Associated With Micronutrients in Breast Cancer Patients During Antineoplastic Treatment	In combination with anti-cancer therapy	Breast cancer	Diet plans and recommendations will be based on the individual’s nutritional status, symptoms, and treatment side-effects; socioeconomic and cultural preferences; as well as the WCRF/AICR guidelines adapting 1.5 g/kg/d of dietary protein, and when required, a caloric restriction (500–1000 kcal/d). Garlic and cruciferous vegetables will be encouraged, as well as 5–9 servings of fruits and vegetables a day. The program will be based on the macronutrient meal equivalent menu method, and standard food servings will be based on the Mexican Food Equivalent System. Breast cancer patients follow-up will be every 2 weeks and a different diet menu will be provided in each session by a specialized dietitian, until 6 months treatment is completed.	Total body weight (time frame: baseline and after the 6 month food-based intervention).	3 years	2015	
NCT02983279	Caloric Restriction Before Surgery in Treating Patients With Endometrial, Prostate, or Breast Cancer:	In combination with anti-cancer therapy	Breast, endometrial, or prostate carcinomas	Dietary counseling, caloric restriction diet. Patients then undergo 25% caloric intake for 3–12 weeks prior to definitive cancer surgery. Patients then undergo 25% caloric intake for 3–12 weeks prior to definitive cancer surgery.	Change in miR-21 expression assessed in serum (time frame: baseline up to 12 weeks).	5 years	2016	
NCT03340935	Safety, Feasibility, and Metabolic Effects of the Fasting-Mimicking Diet (FMD) in Cancer Patients	In combination with anti-cancer therapy	Any malignant neoplasm	Fasting-mimicking diet (or FMD) consisting of a 5-day plant-based, low-calorie (600 Kcal on day 1, followed by 300 KCal/day on days 2 to 5), low-protein, low-carbohydrate diet.	Safety of the fasting-mimicking diet (FMD) in cancer patients.	2 years	2017	
NCT00099151, NCT00427193, NCT00099099	CALERIE: Comprehensive Assessment of Long-Term Effects of Reducing Intake of Energy	Preventive/prospective	-	Caloric restriction. Diet: patients will meet with the registered dietitian to discuss calorie, protein, and fluid needs. The dietitian will calculate calorie needs. Calorie needs will then be reduced to 30%. Protein needs will be estimated based on 0.8 g/kg	-	4 years	2002	
NCT00653484	Energy Balance Interventions for Colorectal Cancer Prevention	Preventive/prospective	Colorectal carcinoma-predisposed healthy overweight or mildly obese individuals	for 12-week energy balance interventions, comprising a physical activity intervention (+2000 kcal/week), a dietary energy restriction intervention (DER) (−2000 kcal/week), or a combined physical activity and DER intervention (+1000/−1000 kcal/week).	- Growth factors (i.e., fasting insulin, c-peptide, IGF-1, IGFBPs, and leptin). - Circulating indicators of inflammation (i.e., c-reactive protein) and oxidative stress (i.e., isoprostanes).	1 year	2008	
NCT00757094	Safety and Feasibility of Fasting While Receiving Chemotherapy	In combination with anti-cancer therapy	Malignant neoplasm	Patients planning to observe fasting while receiving chemotherapy during the month of Ramadan.	Safety of fasting while receiving chemotherapy (time frame: two months).	2 months	2008	
NCT00936364	Short-Term Fasting Prior To Platinum-based Chemotherapy: Feasibility and Impact on Toxicity	In combination with anti-cancer therapy	Histologically confirmed malignancy for which platinum-based chemotherapy on a 21 day cycle or 14 day cycle is being recommended.	Stage I: Patients are assigned to 1 of 4 treatment groups. Group I: Patients fast for 24 h on day −1. - Group II: Patients fast for 48 h on days −2 and −1. - Group III: Patients fast for 72 h on days −3, −2, and −1. - Group IV: Patients undergo a modified 48-h fast with minimal caloric intake on days −2 and −1. Stage II: Patients are randomized to 1 of 2 treatment arms. - Arm I: Patients fast for 72 h on days −2 and on day 1. - Arm II: Patients proceed to chemotherapy without fasting.	Identification of the longest duration of fasting that is safe (time frame: up to 5 years).	11 years	2009	
NCT01304251	Effects of Short-term Fasting on Tolerance to Adjuvant Chemotherapy in Breast Cancer Patients	In combination with anti-cancer therapy	Breast cancer patients	- Short-term fasting (i.e., 24 h before and 24 h after administration of chemotherapy). - Control: 20 breast cancer patients eat according to the current guidelines for healthy nutrition, from 24 h before until 24 h after the beginning of administration of chemotherapy.	Chemotherapy-induced neutropenia (time frame: approximately 126 days).	5 years	2011	- Safdiet et al., aging (Albany NY, 2009) - de Groot et al. doi:10.1186/s12885-015-1663-5.
NCT01559194	A Randomized Comparison of a Low-Fat or Low-Carbohydrate Dietary Pattern for Weight Loss and Impact on Biomarkers Associated With Breast Cancer Risk in Overweight and Obese Premenopausal Women: Lifestyle Eating and Fitness	Preventive/prospective	Breast cancer	- Active comparator: Low-fat diet + exercise. Subjects were educated about a low-fat diet plus exercise and then followed for weight loss. They were also asked to monitor their physical activity by wearing a pedometer and recording the total steps walked every day. Intervention: Behavioral: Low-fat diet plus exercise. - Active Comparator: Low-carbohydrate diet + exercise. Subjects were educated about a low-carbohydrate diet plus exercise and then followed this for weight loss. They were also asked to monitor their physical activity by wearing a pedometer and recording the total steps walked every day. Intervention: Behavioral: Low-carbohydrate diet + exercise.	Number of women who lose weight when following 1 of 2 different calorie-restricted diets (time frame: 18 months).	2 years	2012	doi:10.1089/jwh.2013.4638
NCT01511276	The Effects of Equivalent Weight Loss With or Without Exercise Training on Breast Cancer Risk Biomarkers in Postmenopausal Women: The SHAPE-2 Study	Preventive/prospective	Breast cancer	- Energy-restricted diet according to the national guidelines for healthy nutrition, creating a mean energy deficit of 500 kCal/day. They are asked to keep their habitual sedentary lifestyle. The aim of this group is to lose 5–6 kg of body weight in 14 weeks. - Mainly exercise-induced weight loss. Exercise program consists of 2 h fitness per week, containing endurance and resistance training, as well as 2 h of Nordic walking. Equivalent to an energy expenditure of 350 kCal/day. Along with the exercise program, participants will follow an energy-restricted diet according to the national guidelines of healthy nutrition creating an extra energy deficit of 250 kCal/day.	Serum sex hormone levels (time frame: 21 weeks): estradiol (total, free), estrone, testosterone, sex-hormone-binding globulin.	5 years	2012	doi:10.1186/1471-2407-13-395
NCT01699906	Diet-Induced Weight Loss Reduces Inflammation and Crown-like Structures and Corrects Immune Dysfunction in Subcutaneous Adipose Tissue In Class 2–3 Obese Women: A Pilot Study	Preventive/prospective	Breast cancer	This study will include nutritional and medical evaluation, a 3 day inpatient hospital stay eating a diet providing 50% of what they were taking before starting the study, and then a nutritionally adequate diet that will allow them to lose about 10% of their initial weight within a 7- to 10-week period. They will have about 4–5 g of fat removed by suction through a syringe and a biopsy of the skin in addition to studies of blood and stool samples.	Adipose tissue inflammation via crown-like structures (time frame: 9 weeks). Diet-induced weight loss of 10% body weight will result in reduction in abdominal subcutaneous fat inflammation, as measured by reduction in adipocyte size determined by microscopy and of CLS number in adipose tissue. Reduction in inflammatory gene expression determined by PCR and selected cytokine protein levels. Increased anti-inflammatory lymphocytes determined by immunohistochemistry or by flowcytometry.	2 years	2012	doi:10.1186/s12967-018-1619-z
NCT01754350	Calorie-restricted, Ketogenic Diet. and Transient Fasting versus Standard Nutrition During Reirradiation for Patients With Recurrent Glioblastoma: The ERGO2 Study	In combination with anti-cancer therapy	Recurrent glioblastoma	Calorie-restricted ketogenic diet and transient fasting. On days 1–3 and days 7–9, restriction of carbohydrates to <60 g and of calories to 21–23 kcal/kg per day; on days 4–6, fasting. On days 1–3 and 7–9, restriction of carbohydrates can be supported by the use of drinks provided by “Tavarlin”.	Progression-free-survival (time frame: 6 months).	4 years	2013	
NCT01954836	Short-Term Fasting During Chemotherapy in Patients With Gynecological Cancer: A Randomized, Controlled Cross-over Trial (FIT)	In combination with anti-cancer therapy	Ovarian or breast cancer	Modified fasting with daily caloric intake of <400 kcal by juices starting 36 to 48 h before beginning chemotherapy, and lasting to 24 h after end of chemotherapy, applied in the first half of scheduled 4 or 6 chemotherapy cycles.	Quality of life, modified FACT-O (time frame: 24 h and 7 days after chemotherapy cycle).	3 years	2013	Bauersfeld et al. doi:10.1186/s12885-018-4353-2.
NCT01886677	Exploring the Impact of Negative Energy Balance in Men With Prostate Cancer	In combination with anti-cancer therapy	Prostate cancer	Both arms will receive the same intervention: a healthful diet plus exercise intervention to promote a weight loss of up to 2 pounds/week. The only difference is the timing of the delivery of the intervention (immediate vs. delayed).	Changes in tumor proliferation rate (Ki-67) over the presurgical study period (minimum of 3.5 weeks, up to 24 weeks) will be explored and compared between the intervention and wait list control arms.	1 year	2013	doi:10.1186/s12885-016-2075-x
NCT02126449	Dietary Restriction as an Adjunct to Neoadjuvant Chemotherapy for HER2-Negative Breast Cancer (DIRECT)	In combination with anti-cancer therapy	Breast cancer patients	FMD	- The percentage of patients with grade III/IV toxicity according to the National Cancer Institute Common Terminology Criteria for Adverse Events (NCI CTCAE) version 4.03. (time frame: 2 years). - The percentage of pCR. (time frame: 4 years).	4 years	2014	
NCT02224807	Exploring Effects of Weight Loss on Ductal Carcinoma In Situ	In combination with anti-cancer therapy	Breast cancer patients	- Active comparator: Progressive resistance training (PRT) and a healthy diet. PRT will be done with resistance bands; participants will receive instruction on three resistance band exercises (triceps, biceps, and shoulder overhead) from an American College of Sports Medicine (ACSM) certified exercise specialist. Participants will also receive dietary counseling from a registered dietitian on correcting nutrient deficiencies that are detected during analysis of their 2-day dietary recalls. - Experimental: PRT and a healthy diet plus weight loss. This arm will receive all components of the active comparator arm, plus counseling to achieve a weight loss of 1.5–2 pounds/week. Participants will be trained on how to achieve this caloric deficit through both dietary restriction and increased physical activity. Weight loss will be promoted via a healthy, nutritionally adequate diet consistent with American Cancer Society guidelines. Protein levels will be based on 0.8 g/kg body weight. The distribution of food groups will be customized for preferences. An exercise program will be tailored taking into account kcal expenditure for various activities at a specific body weight; expenditures of 200–400 kcal/day will serve as a goal. Aerobic training of large muscles (legs) will be emphasized to achieve a greater kcal deficit; ramping of intensity and volume over time will be pursued as per the ACSM guidelines. Participants will train once weekly while supervised by an exercise physiologist and daily at home. Intervention: Behavioral and experimental: PRT and a healthy diet, plus weight loss.	Tumor proliferation (time frame: baseline to time of surgery): Ki67. Weight (time frame: baseline to time of surgery). Feasibility (time frame: baseline to time of surgery): enroll 40 subjects in 2-year study, retain >80% of the sample and complete > 70% of contact sessions.	4 years	2014	doi:10.1016/j.jand.2018.08.164
NCT02449148	Healthy Nutrition and Energy Restriction as Cancer Prevention Strategies: A Randomized, Controlled Intervention Trial	Preventive/prospective	-	Three arms: - 2 days per week fasting with 25% energy intake and 5 days per week at 100% energy intake. - Daily energy intake of 80%. - No intervention: general advice on healthy nutrition.	Changes in gene expression in subcutaneous adipose tissue measured by whole genome sequencing (time frame: assessments at baseline (week 0), and after the intervention phase (week 13)).	2 years	2015	doi:10.1093/ajcn/nqy196
NCT02940470	Weight Loss Pilot Study in Postmenopausal Breast Cancer Survivors	Preventive/prospective	Breast cancer survivors	- Calorie-restricted diet plus exercise. Daily meals plus exercise providing 1000 kcal restriction per day for 12 weeks.	- Change in body weight (time frame: 0, 6, 12, 18 weeks). - The primary objective of this pilot study is to determine the effect of weight loss on a wide range of biomarkers associated with risk of breast cancer recurrence in overweight and obese breast cancer survivors. We hypothesized that weight loss would result in a statistically significant improvement in biomarkers associated with risk of breast cancer recurrence.	2 years	2016	doi:10.1186/s40814-017-0160-9
NCT01175837	Short-Term Fasting Prior to Systemic Chemotherapy: A Pilot Feasibility Study	In combination with anti-cancer therapy	Malignant neoplasm	Cohort I: Patients fast 24 h before day 1 of course 2 of chemotherapy. If fast is well tolerated, patients may escalate fasting by 12 h for each subsequent course of chemotherapy for up to 3 courses in the absence of unacceptable toxicity. Cohort II: Patients fast at the longest fasting regimen found to be safe and tolerable in cohort I before day 1 of each course of chemotherapy for up to 4 courses in the absence of unacceptable toxicity.	- Number of patients hospitalized during fasting period (for reasons that are not attributed to disease or postoperative complications) (time frame: up to 48 h). - Number of patients experiencing greater than or equal to grade 3 adverse event related to the fasting period (time frame: up to 48 h). - Percentage of patients able to achieve designated fasting regimen (i.e., greater than or equal to 50%) (time frame: up to 48 h).	3 years	2010	
NCT02286167	The Feasibility and Biologic Effect of a Modified Atkins-based Intermittent Fasting Diet in Patients With Glioblastoma (GBM)	In combination with anti-cancer therapy	Glioblastoma multiforme	Modified Atkins diet	Percent of patients able to remain on the diet and achieve nutritional goals as defined by cumulative assessment of diet records collected at weeks 4, 6, and 8 with a 60% completion defined as a positive results	5 years	2014	
NCT03795493	Diet Restriction and Exercise-Induced Adaptations in Metastatic Breast Cancer	In combination with anti-cancer therapy	Breast cancer patients, Stage IV or metastatic	- Short-term diet and exercise intervention. Participants assigned to the intervention group will perform both the diet and acute exercise interventions. The interventions will be applied prior to up to six chemotherapy treatments of a consistent protocol. The total number of treatments of a given protocol received prior to treatment conclusion is dependent on patient condition and oncologic care preferences.	Tumor size (time frame: 0–6 weeks before the first chemotherapy treatment and 1–4 weeks after the last chemotherapy treatment).	3 years	2018	
NCT03813381	The Impact of a Moderate Calorie and Protein Restriction Program (CARE-PRO) as an Efficient and Affordable Therapeutic Strategy in Patients With Barrett’s Esophagus	Preventive/prospective	Barret’s esophagus	Calorie restriction will be up to 600 kcal below patients’ energy requirements and the amount of protein will be 0.8 g of protein/Kg body weight, mostly form plant-origin food.	Body weight change (time frame: baseline and after 24 months). A 7% weight loss.	1 year	2019	
NCT02035631	Prevention of Breast Cancer Recurrence Through Weight Control, Diet, and Physical Activity Intervention	Preventive/prospective	Recurrent breast cancer I, II, IIIA (or T1-3, N0–N2, M0)	- Behavioral: Diet. The dietary component, aimed to reduce calorie intake according to individual requirements, will be structured in 1 h weekly sessions led by trained nutritionists. Sessions will concentrate on teaching participants about food groups, the food pyramid, and the Mediterranean diet, as well as how to choose, prepare, and cook hypo-caloric meals. - Behavioral: Physical activity. The physical activity component will include two sessions per week led by trained physical activity monitors, including aerobic exercise of high/moderate intensity and instruction about the at-home exercise activities (3 more sessions).	Time between recruitment date and local and distant recurrence date or end of the 5-year follow-up (whichever occurs first).	8 years	2014	
NCT02792270	Effects Of Caloric Restriction On Post-Operative Complications In Sarcoma Patients Treated With Pre-Operative Radiation Therapy	In combination with anti-cancer therapy	Sarcoma	Caloric restriction diet: patients will meet with the registered dietitian to discuss calorie, protein, and fluid needs. The dietitian will calculate calorie needs. Calorie needs will then be reduced to 30%. Protein needs will be estimated based on 0.8 g/kg BW and then reduced by 70%. Dietitian will educate participants on electrolytes and fluid intake based on the reduced food intake.	- Change in the rate of physical function (time frame: baseline, 6 week, 3 month, and 6 month visits after surgery): Musculoskeletal Tumor Society rating scale (MSTS, a clinician-rated scale scoring). - Change in the rate of physical function (time frame: baseline, 6 weeks, 3 months, and 6 month visits after surgery): Toronto extremity salvage score (TESS, a patient-reported questionnaire scoring.	3 years	2016	
NCT01802346	A Randomized, Phase II Clinical Trial of a Controlled Diet Prior to Selected Chemotherapy Treatment in Breast and Prostate Cancer to Evaluate the Impact on Toxicity and Efficacy	In combination with anti-cancer therapy	Breast cancer; hormone-resistant or recurrent prostate cancer	Low-calorie diet: patients eat a special low-calorie diet during 3 days prior to chemotherapy, during the 12 weeks of chemotherapy, and 2 days after chemotherapy. Patients are provided with all meals and all food to be consumed and maintain a diary of the food consumed and appropriate amounts.	Rate of chemotherapy-related toxicity (time frame: up to 12 weeks). Occurrence of grade 2+ non-hematologic symptomatic toxicity (fatigue, nausea and vomiting, anorexia, neuropathy, mucositis, cystitis, stomatitis), evaluated according to Common Terminology Criteria for Adverse Events version 4.0. The two arms will be compared, in terms of the proportion of patients with the occurrence of one of these toxicities.	7 years	2013	
NCT02710721	Fasting and Nutritional Therapy in Patients With Advanced Metastatic Prostate Cancer	In combination with anti-cancer therapy	Prostatic neoplasms	Patients realize a 60 h modified fast (36 h before and 24 h after chemotherapy) with a dietary energy supply 350–400 kcal per day with fruit and vegetable juices, or if not feasible, an established fasting-mimicking diet of 600–800 kcal according to Longo et al. Between chemotherapy, a Mediterranean diet will be practiced with nutrition training individually and in small groups by trained nutritionists at the study center. Controls: Mediterranean diet.	FACT-P/Taxane/An sum score (time frame: assessment day 0 (baseline) and 7 days after each of 6 chemotherapies (study weeks 1,4,7,10,13,16)), summarized change of FACT score from baseline to day 8 after each chemotherapy session.	3 years	2016	
NCT03162289	Intermittent Fasting Accompanying Chemotherapy in Gynecological Cancers (FIT2)	In combination with anti-cancer therapy	Ovarian or breast cancer	- Fasting patients follow a modified fasting regime of 60–72 h (36–48 h before and 24 h after chemotherapy (CT) with a dietary energy supply of 350–400 kcal per day with vegetable juices during the first four cycles of CT. During the rest of the CT cycles, they will observe two days of caloric restriction (24 h before and after CT). Between CTs, a mainly vegetarian diet will be performed and the patients are encouraged to follow a pattern of time-restricted feeding with 14 h fasting overnight for at least six days a week. The patients will receive individual nutrition training by trained nutritionists. - Control patients follow a 60–72 h vegan diet with sugar restriction (36–48 h before and 24 h after CT) during the first four cycles of CT. During the rest of the CT cycles, they will observe two days of vegan- and sugar-restricted diet (24 h before and after CT). Between CTs, a mainly vegetarian diet will be performed. The patients will receive individual nutrition training by trained nutritionists.	FACT-G (time frame: date of inclusion (baseline), day −2 and +7 at each chemotherapy (CT) in triweekly cycles/−2 days at each CT in weekly cycles; and +7 after the last weekly CT, 4 months after inclusion, 3 weeks after end of CT, and 1, 2, and 3 years after inclusion).	4 years	2017	
NCT03131024	The Effects of Short-term Exercise or Caloric Restriction on Anthracycline Chemotherapy-Related Treatment Toxicity	In combination with anti-cancer therapy	Breast cancer patients, stage I–III	- Dietary supplement: 50% caloric restriction. Meals mimicking participant dietary preferences and matching North American macronutrient guidelines will be provided, consisting of 50% of total caloric intake for 48 h. - Other: Aerobic exercise. The supervised exercise session will consist of a 10 min warm-up, 30 min performed at 70–75% of heart rate reserve, which corresponds to a vigorous intensity, followed by a 5 min cool down.	Change in left ventricular ejection fraction reserve (peak exercise—rest) (time frame: 3–14 days before first anthracycline treatment, 2–3 weeks after completion of anthracycline treatment, one year after initiation of anthracycline treatment).	3 years	2017	
NCT03160599	Restricted Calorie Ketogenic Diet as a Treatment in Glioblastoma Multiforme: A Clinical Study	In combination with anti-cancer therapy	Glioblastoma multiforme	Ketogenic diet will consist of 4:1–1:1 fat/protein + carbohydrate. Carbohydrate is limited to 10–30 g/day. The diet will be supplemented with vitamins, calcium, phosphorus, zinc, and selenium supplements to meet the requirements of U.S. Dietary Reference Intakes (DRI) standard. The basis of dietary design is 70–85% of individual’s total calories. The total calorie intake is based on patient’s activity level and their basal metabolism values, which is obtained from indirect calorimetry or Harris–Benedict formula.	Adverse events of patients on high-fat diet (time frame: 2 years).	2 years	2017	
NCT03700437	Randomized, Controlled Pilot Study to Evaluate Fasting-Mimicking Diet in Patients Receiving Chemo-immunotherapy for Treatment of Metastatic Non-Small Cell Lung Cancer	In combination with anti-cancer therapy	Non-Small Cell Lung Cancer	Chemolieve^®^, a plant-based FMD that provides ~300 calories/fasting day and includes all the food to be consumed during the dietary intervention, including supplements. Subjects will start the diet 3 days prior to chemo-immunotherapy and continue on the first day of chemo-immunotherapy for the first 4 cycles of therapy.	To determine the effect of fasting-mimicking diet (FMD) on circulating tumor cells (CTCs) in patients with advanced NSCLC receiving chemo-immunotherapy.		2018	
NCT03595540	Phase II Clinical Study of a Fasting-Mimicking Diet in Patients Undergoing Oncologic Treatment	In combination with anti-cancer therapy	Breast and colorectal cancer	Prolon FMD	- Percentage of prescribed diet consumed and intake of any extra food (time frame: 6 months). - Quantification of FMD-emergent adverse events (time frame: 6 months), according to NCI CTCAE 5.0.	2 years	2018	
NCT02379585	A Pilot Study of Short-Term Fasting on Neoadjuvant Chemotherapy in Patients With Newly Diagnosed Breast Cancer (STEFNE Study)	In combination with anti-cancer therapy	HER2-positive breast cancer	Patients will fast 24 h before and 24 h after the administration of chemotherapy.	Pathological response rate at the time of surgery or at the time of biopsy (time frame: 4–6 cycles (up to 12 weeks)).	2 years	2015	
NCT00467220	Effect of Daily Calorie Restriction or Alternate-Day Reductions in Calorie Intake on Risk for Cardiovascular Disease and Cancer	Preventive/prospective	-	- Alternate day fasting arm: Subjects in this arm will be asked to alternate between one day of eating as they wish versus one day on a calorie-restricted meal plan. Subjects will follow this alternating meal plan for 3 months. - Calorie restriction: subjects in this arm will be asked to follow a calorie-restricted meal plan, daily, for three months.	Adipose tissue dynamics (triglyceride turnover, lipolysis, de novo lipogenesis, adipose cell proliferation), adipose tissue morphology (cell size and number), adipose tissue hormone levels (adiponectin, leptin), skin turnover (keratin dynamics), T-lymphocyte proliferation, as well as plasma lipid and lipoprotein, homocysteine, and C-reactive protein levels.	10 years	2007	
NCT02607826	Short-Term Starvation vs. Normal Diet Before Chemotherapy of Solid Tumors	In combination with anti-cancer therapy	Cholangiocarcinoma pancreatic ductal adenocarcinoma Colorectal cancer Gastric cancer Adenocarcinoma of the esophagogastreal Junction esophagus cancer	Short-term starvation for a timeframe beginning 24 h prior to chemotherapy administration, lasting until 6 h after administration.	Primary endpoint of this study is to assess the improvement in response to therapy for patients undergoing short-term starvation before chemotherapy of solid tumors in comparison to patients without dietary restrictions. Response to therapy on MRI or CT scans will be measured using the RECIST criteria version 1.1.	4 years	2015	
NCT02960711	Randomized, Controlled Trial of Metformin and Dietary Restriction to Prevent Age-Related Morbid Events in People With Metabolic Syndrome	Preventive/prospective	Any malignant neoplasm	- Experimental: Metformin (1700 mg/day) + Lifestyle Metformin: 2 tablets per day, one at breakfast (or lunch) and one at dinner, of either metformin (two 850 mg tablets/day) + participation in the lifestyle intervention activities. Intervention: Drug: Metformin hydrochloride 850 mg oral tablet (Glucophage). Placebo comparator: placebo + lifestyle. Placebo: (two identical tablets) according to the blind assignment + participation in the lifestyle intervention activities. Intervention: Drug: Ludipress, magnesium stearate, micronized hydrated silica, talcum. - Experimental: Metformin (1700 mg/day) alone. Metformin: 2 tablets per day, one at breakfast (or lunch) and one at dinner, of metformin (two 850 mg tablets/day). Intervention: Drug: Metformin hydrochloride 850 mg oral tablet (glucophage). Placebo comparator: placebo alone. Placebo: (two identical tablets) according to the blind assignment. Intervention: Drug: Ludipress, magnesium stearate, micronized hydrated silica, talcum.	Total incidence of age-related chronic diseases (time frame: 5 years). Records for all age-related chronic diseases, but first concentrate the analysis on cancer, coronary heart disease, stroke, and diabetes.	2 years	2016	doi:10.5301/tj.5000599
NCT01784042	Effect of Dietary Energy Restriction and Omega-3 Fatty Acids on Mammary Tissue and Systemic Biomarkers of Breast Cancer Risk	Preventive/prospective	Breast cancer patients	Lovaza (omega-3-acid ethyl esters) +/– dietary energy restriction.	Ki67 expression by hyperplastic breast lesions.	4 years	2013	
NCT00470119	Effect of a Low-Calorie Diet and/or Exercise Program on Risk Factors for Developing Breast Cancer in Overweight or Obese Postmenopausal Women	Preventive/prospective	Breast cancer	- Caloric restriction. Nutritionist-delivered weight loss intervention though diet modification with an aim of 10% weight loss over a year-long intervention based on the DPP and LookAHEAD interventions. Participants meet with a nutritionist individually and in small groups. Participants receive general information about diet and behavior strategies, such as self-monitoring, goal-setting, stimulus control, problem-solving, and relapse prevention training. Participants learn to set a calorie goal and a fat gram goal, and how to achieve the goal calorie reduction. Meetings are held weekly during the first 6 months of the diet program but taper off over the course of the study. - Exercise intervention participants exercise 3 days per week under the supervision of a physiologist and 2 days per week independently at home, for a total of 5 exercise sessions (at least 45 min of moderate-intensity exercise per session) weekly over 12 months. - Caloric restriction and exercise intervention. Combined caloric restriction and exercise intervention.	Serum estrone concentrations as measured by radioimmunoassay (time frame: at baseline and 12 months timepoint).	5 years	2007	doi:10.1249/MSS.0000000000000480
